# Tunable one-dimensional electron gas carrier densities at nanostructured oxide interfaces

**DOI:** 10.1038/srep25452

**Published:** 2016-05-06

**Authors:** Houlong L. Zhuang, Lipeng Zhang, Haixuan Xu, P. R. C. Kent, P. Ganesh, Valentino R. Cooper

**Affiliations:** 1 Center for Nanophase Materials Sciences, Oak Ridge National Laboratory, Bethel Valley Road, Oak Ridge, Tennessee 37831, United States; 2 Department of Materials Science and Engineering, The University of Tennessee, Knoxville, Tennessee 37996, United States; 3 Computer Science and Mathematics Division, Oak Ridge National Laboratory, Bethel Valley Road, Oak Ridge, Tennessee 37831, United States; 4 Materials Science and Technology Division, Oak Ridge National Laboratory, Bethel Valley Road, Oak Ridge, Tennessee 37831, United States

## Abstract

The emergence of two-dimensional metallic states at the LaAlO_3_/SrTiO_3_ (LAO/STO) heterostructure interface is known to occur at a critical thickness of four LAO layers. This insulator to-metal transition can be explained through the “polar catastrophe” mechanism arising from the divergence of the electrostatic potential at the LAO surface. Here, we demonstrate that nanostructuring can be effective in reducing or eliminating this critical thickness. Employing a modified “polar catastrophe” model, we demonstrate that the nanowire heterostructure electrostatic potential diverges more rapidly as a function of layer thickness than in a regular heterostructure. Our first-principles calculations indicate that for nanowire heterostructures a robust one-dimensional electron gas (1DEG) can be induced, consistent with recent experimental observations of 1D conductivity at LAO/STO steps. Similar to LAO/STO 2DEGs, we predict that the 1D charge density decays laterally within a few unit cells away from the nanowire; thus providing a mechanism for tuning the carrier dimensionality between 1D and 2D conductivity. Our work provides insight into the creation and manipulation of charge density at an oxide heterostructure interface and therefore may be beneficial for future nanoelectronic devices and for the engineering of novel quantum phases.

The unique physical properties of oxides, such as high dielectric constants and possible couplings between electric, magnetic and elastic degrees of freedom have long been explored for use in sensors and low power-high speed memories. Emergent phenomena, such as the appearance of a two-dimensional electron gas (2DEG) at oxide heterointerfaces^1^,^2^,^3^ have created the opportunity for application in transistors^3^,^4^,^5^,^6^,^7^,^8^,^9^,^10^ in addition to providing a platform for studying the confinement of electrons in two dimensions. For example, the prototypical heterostructure, LaAlO_3_(LAO)/SrTiO_3_(STO), exhibits a wealth of intriguing properties at the interface such as metallicity^11^,^12^, magnetism^13^, and novel quantum phases such as superconductivity^14^ that are absent in the parent compounds.

The emergence of a 2DEG in LAO/STO can be understood through the “polar catastrophe” model illustrated in Fig. 1(a,d)^15^,^16^,^17^. In an unreconstructed superlattice, the electrostatic potential diverges as the number of LAO layers increases due to the alternating nominal + 1/− 1 charges in the LaO and AlO_2_ layers, respectively. At roughly 4 layers, when the magnitude of the divergent potential is comparable to the STO band gap, half of an electron per AlO_2_ surface layer is transferred to the interface (see Fig. 1(d)) in order to compensate for the large surface potential^3^. Surprisingly, recent experiments demonstrate that this critical thickness can be significantly altered through nanostructuring; thereby producing interfacial conductivity below the 4 LAO layers required for the “polar catastrophe” mechanism. Here, the LAO nanowire was comprised of alternating one and three LaAlO_3_ unit cell nanowires, fabricated by standard lithography techniques, and thought to result in conducting one-dimensional channels at the boundary between the 1 and 3 layer LAO step^18^.

Stimulated by this experimental study, we investigate a nanowire heterostructure model formed by placing a LAO nanowire on top of a STO (001) substrate. The stepped LAO surface can be regarded as consisting of adjacent one and *m* LAO layers, each 2 unit cells wide, repeated in one lateral direction (see Fig. 2). Therefore, we henceforth refer to this interface as a 1/*m* nanowire heterostructure; where a 1/3 nanowire is similar to the experimental setup of Ron *et al.*^18^. Similarly, a regular interface, *e.g.*, four LAO layers on a STO substrate would be denoted as a 4/0 interface. We emphasize that our nanowire model differs from traditional heterostructures due to the imbalance in the layer-by-layer charge. As such, our model serves as the first prototype for understanding emerging nanowire heterostructures.

In this article, we explore the electronic structure origins of metallicity in stepped nanostructured LAO/STO interfaces (nanowires) using first-principles density functional theory. We demonstrate that signatures of a confined electron gas appear below the aforementioned threshold number in these nanostructures. To explain this phenomenon, we invoke a modified polar catastrophe model to understand the occurrence of metallic states in these nanowire heterostructures. Our results indicate that the larger charge difference between the La_3_O_2_ and Al_2_O_5_ layers in the non-stoichiometric nanowires causes a faster buildup of potential across the slab, thereby driving this charge transfer below the expected 4 layers. Interestingly, our results suggest the presence of tunable 1D conduction channels due to the nanowire geometry. Also, we find that because of the fact that the La_3_O_2_ and Al_2_O_5_ layers no longer have a + *n*/− *n* charge balance, as in traditional heterostructures, the potential across the slab continues to grow with increasing layers, resulting in an increase in carrier densities when going from the 1/2 to the 1/3 nanostructures. Furthermore, for 1/*m* nanostructures we find a crossover from 1D conducting channels to more 2D-like carrier densities as m decreases from 3 to 2.

Conversely, we observe no charge transfer in the stoichiometric nanostructures which have a + 1/− 1 charge balance, and in fact the insulator-metal transition (IMT) occurs at a much higher value than 4-layers; closer to 8 layers. Again, this can be understood based on a simple electrostatic model which shows that the interfacial polar discontinuity and hence the interfacial charge density and the corresponding potential is directly proportional to the layer coverage; thus half-coverage leads to a transition occurring at nearly twice the critical thickness of 4 LAO layers found with full LAO coverage. This is consistent with the experimental study on the LAO-STO solid-solution overlayer, where the critical thickness was found to increase inversely proportional to the concentration of LAO in the solid-solution^19^. Ultimately, these results suggest that nanostructuring provides tremendous opportunities for tuning the number and dimensionality of carriers at an oxide interface; which has natural consequences for the design of novel devices based on oxide heterostructures.

## Methods and Computational Details

We performed density-functional theory (DFT) calculations using the projector augmented wave (PAW) method as implemented in the Vienna *Ab-initio* Simulation Package (VASP)^20^,^21^,^22^. The Sr 4*s*4*p*5*s*, Ti 3*p*3*d*4*s*, La 5*s*5*p*5*d*6*s*, Al3*s*3*p*, and O 2*s*2*p* electrons were treated as valence electrons. For all calculations, a cutoff energy of 400 eV was used to expand the electronic wave functions in the plane wave basis set. We used the Perdew-Burke-Ernzerhof (PBE) exchange-correlation functional^23^. To deal with the electron localizations of Ti *d* and La *f* orbitals in the LAO/STO heterostructure, we employed the Dudarev method with a rotationally invariant *U*_eff_ =  *U*-*J* =  7.0 eV and 7.5 eV for Ti *d* and La *f* orbitals, respectively^24^. These parameters have also been used in a previous study^25^. In addition, with the selected *U*-*J* parameters, we confirm that the critical number of LAO layers for the insulator-to-metal transition at bulk interfaces is four, which agrees with previous theoretical studies^26^,^27^.

For all systems we considered 4 in-plane unit cells in the lateral direction along *x* and 1 unit cell in *y* (*i.e.*, along the nanowire) (Fig. 2). The in-plane lattice constants in the *x* and *y* directions were fixed to 4*a*_0_ and *a*_0_ respectively, where *a*_0_ is the calculated PBE+ *U* lattice constant of bulk STO, i.e. *a*_0_ =  3.943 Å. Nine and a half unit cells of STO layers were used to model the STO (001) substrate. Similar to ref. 28, each supercell contained two symmetric surfaces. LAO nanowires were arranged such that the stepped LAO surface had an alternating pattern of two unit cells of one LAO layer and then two unit cells of *m* LAO layers (Fig. 2). A vacuum spacing of ~20 Å was used to ensure that the interactions between the two surfaces were negligible. We considered two types of nanowire structures: non-stoichiometric and stoichiometric. In the nonstoichiometric case, a complete unit cell was generated in the *m* layers of the nanowire, whereas in the stoichiometric case, ions were removed in order to retain charge neutrality by enforcing the LAO formula unit. The *k*-point sampling used the Monkhorst-Pack scheme^29^ and employed a Γ -point-centered 8 ×  8 ×  8 mesh for the bulk calculations and a Γ -point-centered 2 ×  8 ×  1 mesh for the interface calculations. For the band structure calculations, we used 40 *k* points along the high-symmetry paths. All atomic positions were relaxed until the Hellmann-Feynman forces were less than 0.03 eV/Å. To confirm the 1D nature of the electron gas, additional supercells containing 6 unit cells in the *x* direction were studied for both the 1/2 and 1/3 non-stoichiometric nanostructures. In these cases, an alternating pattern of 4 unit cells of 1 layer of LAO and 2 unit cells of *m* layers of LAO were modeled. This geometry was chosen since 4 unit cells is larger than the previously predicted electronic gas screening length of 2–3 unit cells in STO^28^,^30^,^31^,^32^,^33^,^34^. Notably, the largest supercell in this work consists of as many as 396 atoms.

## Results

We start with the discussion of the electronic structure of the non-stoichiometric nanostructures. Figure 3 shows the band structure of the 1/2 and 1/3 non-stoichiometric nanostructures. In both cases, the interfacial Ti *d* bands cross the Fermi level demonstrating the unexpected metallicity. More interestingly, the metallic states of the nanostructures exhibit characteristics of a 2DEG^35^,^36^,^37^,^38^,^39^; as can be seen by the parabolic dispersions around Γ and the flat bands in the Γ -X and Γ -Y (not depicted here) directions. An orbital-resolved analysis shows that the metallic states just below the Fermi level are comprised of the three Ti *t*_*2g*_ *d* bands, i.e. the light Ti *d*_*xy*_ band and the significantly heavier (along Γ -X and Γ -Y) *d*_*xz*_ and *d*_*yz*_ bands. Note that the now occupied Ti *d* bands for the 1/3 non-stoichiometric structures are much lower in energy than those of the 1/2 nanostructure, suggesting a significant change in the carrier densities between the two systems; with more electrons at the interface of the 1/3 nanostructure.

Figure 4 shows the layer averaged spatial distribution of the excess electrons in the TiO_2_ layers in STO obtained by integrating the partial density of states within the energy window between − 1.0 eV and the Fermi level for each Ti atom. First, we see that there is a larger charge accumulation in the Ti layer near the interface in the non-stoichiometric 1/3 layer than in the 1/2 layer. Similar to regular LAO/STO heterostructures^28^,^30^,^31^,^32^,^33^,^34^, the transferred electrons are not strictly confined to the interface, but decay several layers into the STO (001) substrate. A summation of the total electron density gives 0.5 and 1.0 electrons per surface unit cell for the 1/2 and 1/3 nanostructures, respectively. This translates to 0.25 and 0.5 e^−^ per interface unit cell.

To understand the driving force behind the electronic reconstruction in the 1/2 and 1/3 nanostructures, we propose a modified “polar catastrophe” model^15^. In the case of the unreconstructed non-stoichiometric systems, the layers are comprised of La_1.5_O and AlO_2.5_ layers with nominal charges of + 2.5 and − 2 respectively.

**Figure 1:**
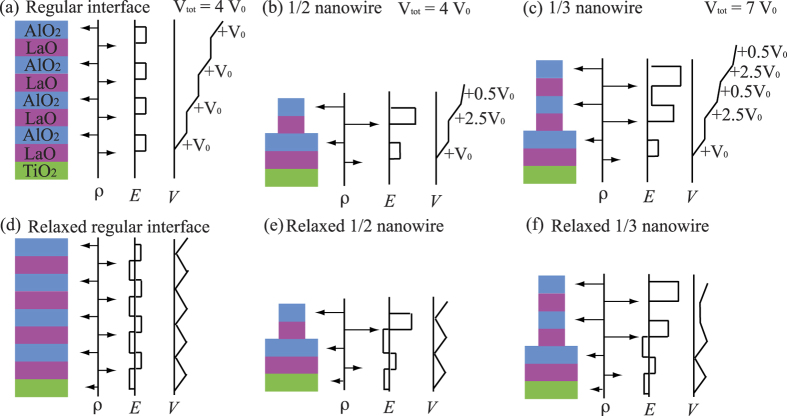
Schematic of charge density (*ρ*), electric field (*E*), and electrostatic potential (*V*) profiles for the unrelaxed (**a**) regular, (**b**) 1/2, (**c**) 1/3 LAO/STO heterostructures. The corresponding profiles for relaxed heterostructures are shown in (**d**–**f** ), respectively.

Even for the 1/2 case (see Fig. 1b,e), this would result in a large enough potential buildup across the LAO layers (comparable to the STO band gap and on the order of that in the standard LAO/STO heterostructures). To compensate for this charge buildup, a simple electrostatic model estimates the need for a total charge transfer of 0.5 e^−^/LAO surface unit cell (or rather 0.25 e^−^/interface unit cell). This estimate is in remarkable agreement with the charge density profile (i.e. the sum of the layer averaged charge densities) depicted in Fig. 4(a). However, due to the fact that the La_1.5_O layer has a much larger absolute charge than the AlO_2.5_ layer, the addition of an additional LAO layer once again would result in a diverging potential (Fig. 1c,f); requiring an additional 0.5 e^−^/LAO surface unit cell to be transferred to the interface. In this context, an electrostatic model would suggest the need for a total of 0.5 e^−^/interface unit cell to compensate for this potential divergence. This is once more consistent with our DFT findings for the 1/3 system. Furthermore, it suggests that the growth of additional layers may be useful in inducing even more charge transfer (electronic reconstruction) and thus could be important for tuning the carrier densities at this interface.

Interestingly, we find that within a layer the distribution of electrons is also not uniform. Figure 5 depicts the excess electronic charge on the Ti atoms at the interface in the 1/2 and 1/3 non-stoichiometric nanowire heterostructures. Here, we see that the ions below the nanowire layers have a larger charge accumulation, which seems to decay as we move away from the interface. A Gaussian fit (Fig. 5 solid black lines) to the data, assuming overlapping density from periodic wire images, suggests that the true system maybe one-dimensional with the typical 3 unit cell spread of electrons away from the nanowire overlayer; as commonly observed in oxide heterostructure 2DEGs^28^,^30^,^31^,^32^,^33^,^34^,^37^,^38^,^39^. This implies the existence of a 1DEG state brought about through the localization of the overlayer potential to just below the nanowire. Our results also suggest that the 1DEG state may be more clearly defined for nanowires separated by more than 2 unit cells; as was done in the recent experiments^18^. Furthermore, we also infer that the electron density at the interface would be tunable through modulation of the separation between the nanowires.

To further examine the 1D nature of the conducting states, we simulated larger supercells with 6 unit cells in the *x* direction, comprised of alternating 4 unit cell-wide steps of 1 layer LAO adjacent to 2 unit cell-wide steps of *m* layers of STO (i.e. a separation of 4 unit cells between nanowires). Indeed, for the 1/3 non-stoichiometric nanowires we find that the charge density is strongly confined to the layers below the 3 LAO step. Again we see that there should be a few unit cells dispersion of carrier density away from the 3 LAO layer step (Fig. 5 top). More interestingly, we see that in the case of the 1/2 layer, there is less confinement of electrons and carriers are now almost evenly smeared across the interface (Fig. 5 bottom). This implies the formation of a 2D electron gas rather than a 1D nanowire. In light of these results, we can reinterpret the previous experimental results as follows: for the 1/3 nanostructure the 1D-like conductivity measured in a similar LAO-STO step^18^ may be due to the large confinement within the nanowire of the electrons transferred from the surface layer of the 3 step LAO nanowire to the interface Ti-*d* orbitals. However, we would expect that for the 1/2 nanostructure, there would be nearly a factor of two drop in the number of carriers inside the nanowire. In the limit of a large nanowire diameter (as opposed to 2 u.c. diameter in our simulation) we therefore predict a weak 2D electron gas. This is consistent with the absence of a 1D-like repeated modulation in the experimental measurement of the conductivity under an applied voltage in recent experiments on the 1/2 stepped structure^40^.

Commensurate with the electronic reconstruction we observe significant atomic displacements away from the interface in the Ti layers in the 1/2 and 1/3 non-stoichiometric 4 unit cell-wide nanostructures. Figure 4(c) depicts the magnitude of the layer-averaged Ti displacements at the TiO_2_ layers near the interface. Furthermore, we find that the displacements in the 1/2 nanowire heterostructure is roughly half of that in the 1/3 case. These displacements are consistent with the notion of polar distortions assisting in charge screening due to electrostriction at the interface and with the idea that larger distortions are linked to larger charge densities at the interface^16^,^17^,^28^,^41^. In particular, the magnitude of the average interfacial displacement in the 1/3 interface is similar to that observed in previous studies of LAO/STO^32^,^33^,^42^,^43^,^44^,^45^ and La δ -doped STO superlattices^37^,^38^,^39^ where the interfacial charge density is also 0.5 e^−^ per interface unit cell. Again, a survey of individual ionic displacements shows signatures of 1D behavior; with larger displacements directly below the nanowire (i.e. on ions with larger charge densities).

Given the large changes in carrier densities at the interfaces, a semiconductor physics-based interpretation would suggest significant changes in mobilities of the electrons at the interface (either due to changes in band effective masses or scattering lengths). To try to understand the effects on changes in carrier densities we evaluated the band effective masses of the two systems. (N.B. Scattering lengths are not accessible from DFT band structure calculations.) For both of the 1/2 and 1/3 nanowire heterostructures, the electron effective masses of the *d*_*xy*_ band at the Γ point are nearly isotropic with 0.53 *m*_*e*_, where *m*_*e*_ is the electron rest mass. In contrast, the electron effective masses of the *d*_*yz*_ and *d*_*xz*_ bands are anisotropic. For example, along the Γ to X direction, the flat *d*_*yz*_ band shows much heavier electron masses of 4.47 and 4.50 *m*_*e*_ for the 1/3 and the 1/2 nanowire heterostructures, respectively. For the Γ to M direction the band effective masses for the *d*_*yz*_ and *d*_*xz*_ are 0.55 and 0.59 *m*_*e*_, respectively. Previous work on La δ -doped STO superlattices and from ARPES measurements on the STO surfaces found band effective masses in the range of 0.50–0.60 *m*_*e*_^35^,^36^,^38^,^39^,^46^. As such, our computed effective masses suggest that the electron mobilities should be comparable to standard LAO/STO heterostructures. It should be noted that recent work on oxide heterostructures suggests that the decoupling of the dopant layer from the charge carrier layers may result in minimal effects of carrier concentrations on scattering lengths in these materials^39^,^47^.

Additionally, it has been shown that the transport in these materials is via a two-carrier model, comprised of high density low mobility carriers (primarily *d*_*xy*_ electrons) and low density high mobility carriers (*d*_*xz*_ and *d*_*yz*_ electrons). Although the *d*_*xy*_ bands have the smallest effective mass, previous effective Hamiltonian studies have demonstrated that, due to orbital ordering, electrons in these bands are strongly localized and therefore are not mobile. Hence, the ratio of the density of these electrons may be useful in understanding the ultimate change in the mobilities in these materials. Therefore, to gain further insight into how the populated *d* (i.e. *d*_*xz*_, *d*_*yz*_ and *d*_*xy*_) orbitals affect the carrier mobility of the 1/2 nanowire heterostructure. We calculate the fractional occupation *f*, which is defined as^37^,


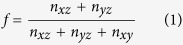


where *n*_*xz*_, *n*_*yz*_, and *n*_*xy*_ are the number of electrons in the *d*_*xz*_, *d*_*yz*_ and *d*_*xy*_ orbital, respectively. The calculated *f* for each TiO_2_ layer is displayed in Fig. 4(b). Again, we see that there is very little difference in *f* between the two heterostructures. These results agree with previous results for the La δ -doped system^37^, again suggesting little changes in mobilities in these materials^39^,^47^.

Although the computed electron count (see Fig. 4(a)) at the interface is larger than typically observed in experiment, the number of mobile carriers is significantly less, i.e. only one tenth of the total *d* electrons. We therefore expect to obtain a combination of high density low mobility carriers and low density high mobility carriers-the latter of which are usually seen in transport measurements.

**Figure 2:**
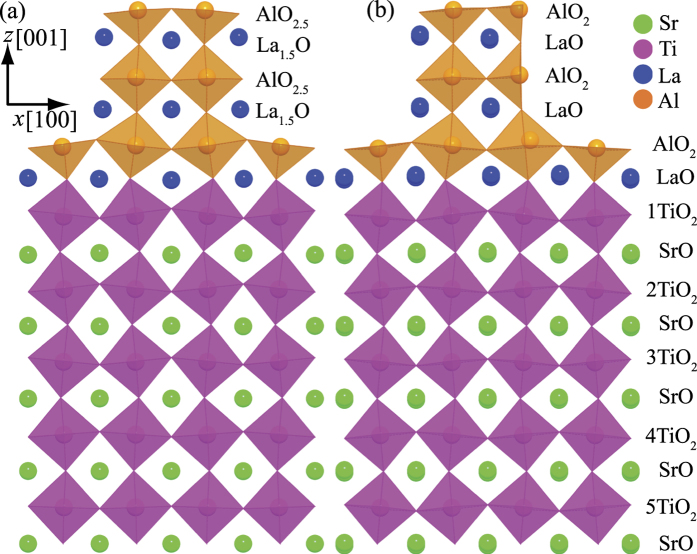
Relaxed (**a**) non-stoichiometric and (**b**) stoichiometric 1/3 LAO/STO nanowire heterostructure. Only one half of the symmetric slab in each supercell is shown. Ti- and Al-centered octahedra are represented by magenta and orange polyhedra, respectively. The numbers before the TiO_2_ notations denote the TiO_2_ layer number relative to the LAO/STO interface.

**Figure 3:**
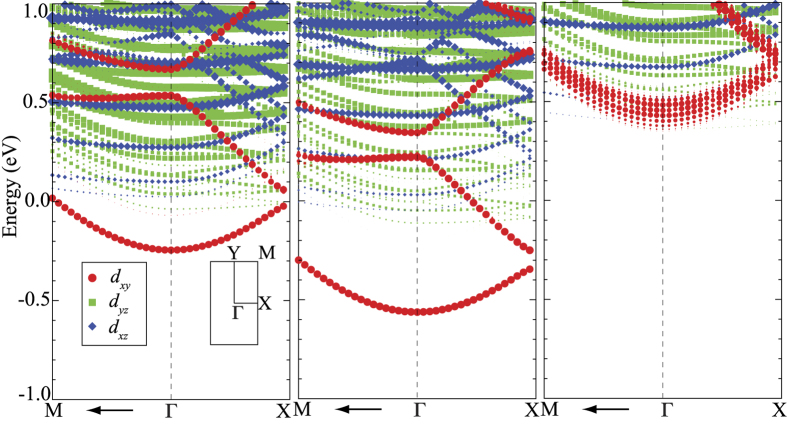
The orbital-resolved interfacial Ti d-bands for the (Left) 1/2 non-stoichiometric, (Middle) 1/3 non-stoichiometric and (Right) 1/3 stoichiometric LAO/STO nanostructures.

**Figure 4:**
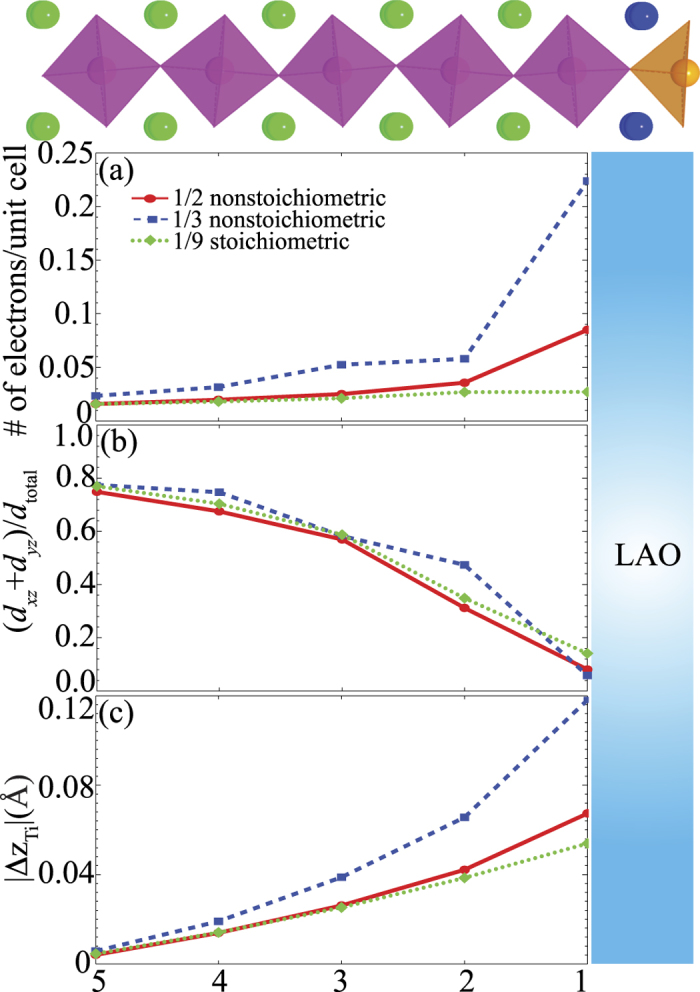
Layer averaged (**a**) charge density profiles, (**b**) *d* orbital occupation fractions, and (**c**) off-center atomic displacements as a function of relative c-axis coordinate of Ti for various LAO/STO nanowire heterostructures.

**Figure 5:**
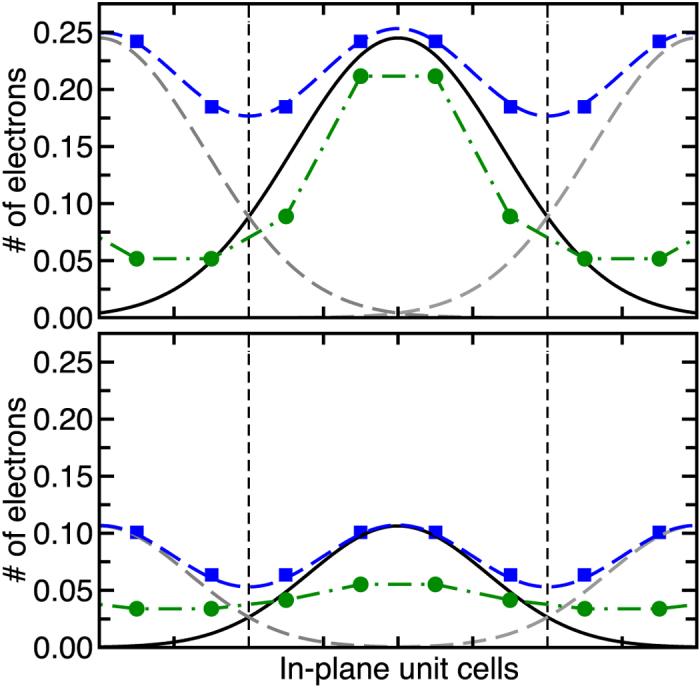
Interfacial Ti atoms electron count for the 1/3 (top) and 1/2 (bottom) nanostructures. Blue squares denote computed electron count for individual interfacial Ti atoms. Solid black and dashed gray curves denote Gaussian fits to data assuming overlapping charge from neighboring wires. Dashed blue line denotes sum of Gaussians indicating match with computed DFT charge distribution. Green lines indicate electron counts from 6 unit cell wide nanostructures.

A final point to consider is the atomic and electronic structures of a stoichiometric nanostructure. Figure 2(b) shows the relaxed configuration of the stoichiometric 1/3 nanowire heterostructure. Similar atomic displacements are observed as in the non-stoichiometric 1/3 case. However, in this case we do not observe any significant electronic reconstruction at the interface. The reason for this lies in the fact that in the stoichiometric case, the alternating LaO and AlO_2_ layers have alternating + 1 and − 1 charges but with dimensions of only half of a layer; and therefore the potential across these slabs do not grow as quickly as in the non-stoichiometric case. By increasing the number of *m* stoichiometric LAO layers, we observe a critical thickness of 1/9 LAO layers for the interface to become marginally metallic. This can be seen from the charge profile shown in Fig. 4. In addition, the fractional occupation and the Ti off-center displacements are now similar to the 1/2 and 1/3 non-stoichiometric nanowire heterostructures. Here, the delayed crossover draws analogy with the experimental study on LAO-STO solid-solution overlayers where the nanowire LAO can be thought of as a 50/50 LAO-STO composition^19^. In this instance, the critical thickness was found to also double in experiment. It is important to note that even for thinner steps, such as 1/3 stoichiometric nanowires, we observe little difference in the band structures of the Ti *t*_*2g*_ conduction states. This suggests that even in the cases where a gate voltage is applied to inject electrons into the system that the confinement of electrons within the 1D states should be similar. Nevertheless, these results hint at the fact that the control of stoichiometry may provide an alternative route for tuning the electronic properties of the LAO/STO heterostructures.

## Conclusion

In conclusion, we have computationally examined a novel LAO/STO nanostructure. Our results suggest that for non-stoichiometric, fully oxidized nanowires, this architecture should give rise to a 1DEG with mobilities that are comparable to those in standard LAO/STO and La δ -doped systems. Moreover, we demonstrate that these δ -doped systems result in the emergence of interfacial metallic states well below the LAO/STO critical thickness of 4 LAO layers. This is the first time that a true 1D electron density profile is achieved by tuning the fundamental length scales for separation and thickness of the nanowires. In addition, we observe a fascinating phenomenon that the electrostatic potential continues to diverge and remains localized. To understand this observation, we invoke a modified “polar catastrophe” model demonstrating that, due to the larger layer-by-layer charges in the non-stoichiometric layer, the transfer of charge to the interface should occur for 1/2 nanowires with roughly 0.5 e^−^/per surface LAO transferred to the interface. Furthermore, the imbalance in layer-by-layer charge drives further movement of electrons to the interface, producing twice as many electrons at the interface for a second LAO nanowire layer. Our model establishes fundamental theoretical underpinnings for the tunability of conduction in oxide nanowires and provides novel insights into defects at the edge of wires that would lead to either charge imbalance and/or stoichiometric layers. Similar to experiments for LAO-STO solid solution overlayers, this charge transfer is delayed for stoichiometric layers due to the smaller effective concentration of LAO on the surface. Band effective mass and orbital fractional occupation analyses show that these values are very similar to previous observations for La δ -doped superlattices, indicating the possibility of similar mobilities in these materials. Given the nature of the emergent 1DEG interfacial states, these findings should be generalizable to other similar oxide nanowire heterostructures and for other materials combinations. These results provide useful insights into the origin of the 1DEG recently found in experiment^18^. Most importantly, we demonstrate how carrier dimensionality and densities can be modified through nanostructuring and precise control of stoichiometry, thereby identifying new opportunities for understanding and controlling the properties of electronic states at oxide heterostructure interfaces.

## Additional Information

**How to cite this article**: Zhuang, H. L. *et al.* Tunable one-dimensional electron gas carrier densities at nanostructured oxide Interfaces. *Sci. Rep.* **6**, 25452; doi: 10.1038/srep25452 (2016).
